# Psychosocial burden and associated factors among nurses in care homes during the COVID-19 pandemic: findings from a retrospective survey in Germany

**DOI:** 10.1186/s12912-022-00807-3

**Published:** 2022-02-10

**Authors:** Christian Hering, Annabell Gangnus, Andrea Budnick, Raphael Kohl, Elisabeth Steinhagen-Thiessen, Adelheid Kuhlmey, Paul Gellert

**Affiliations:** 1 Institute of Medical Sociology and Rehabilitation Science, Charité – Universitätsmedizin Berlin, Germany, Virchowweg 22, 10117 Berlin, Germany; 2 Deparment of Endocrinology, Diabetes and Metabolism, Charité – Universitätsmedizin Berlin, Berlin, Germany

**Keywords:** Nurses, Care homes, Nursing homes, COVID-19, Psychosocial burden, Depression, Anxiety, Stress, SARS-CoV-2

## Abstract

**Background:**

Care homes were hit hard by the COVID-19 pandemic. Although high levels of psychosocial burden (i.e., anxiety, depression and stress) during the pandemic have been described for healthcare workers in hospitals, evidence on the psychosocial burden for nurses in care homes during the pandemic is scarce.

**Methods:**

A total of 811 nurses participated in a retrospective online survey between November 2020 and February 2021. Information about the COVID-19 situation (i.e., working demands, COVID-19 cases in their facility, and COVID-19-related burden) of nurses in German care homes during the first wave of the pandemic (March 2020 to June 2020) was gathered. The Stress Scale of the Depression Anxiety and Stress Scales (SDASS-21), the Generalized Anxiety Disorder Scale-2 (GAD-2), the Patients-Health-Questionnaire-2 (PHQ-2), and the Copenhagen Psychosocial Questionnaire (COPSOQ) were used to screen for psychosocial burden.

**Results:**

Among nurses, 94.2% stated that working demands since the COVID-19 pandemic increased. Further, 59.1% showed clinically relevant levels of either stress, anxiety, and/or depression. Multiple regression analysis showed significant associations between COVID-19-related burden and qualification (*p* < .01), dissatisfaction with COVID-19 management of care home manager (*p* < .05), COVID-19-related anxiety (*p* < .001), and dementia as a focus of care (*p* < .05). Stress, depression, and anxiety showed associations with COVID-19 related burden at work (*p* < .01), COVID-19-related anxiety (*p* < .001), social support (*p* < .01), and sense of community (*p* < .05). Stress was also associated with COVID-19 cases among residents (*p* < .05), and size of care home (*p* < .05).

**Conclusion:**

Short- and long-term strategies (i.e., psychosocial counseling, mandatory team meetings, more highly qualified nurses, additional training) in the work environment of nursing, in crises, but beyond, should be encouraged to reduce the burden on nursing staff in care homes.

**Supplementary Information:**

The online version contains supplementary material available at 10.1186/s12912-022-00807-3.

## Background

The World Health Organization (WHO) declared the spread of the novel Coronavirus (SARS-CoV-2) as a pandemic on the 11^th^ of March, 2020 [1]. Care homes were hit hard by the pandemic, especially during the first and second pandemic waves. By February 2021, approximately 41% of COVID-19-deaths worldwide were care home residents [2]. In Germany, the estimated share of 50% of all deaths due to COVID-19 was in care home residents only during the first wave of the pandemic [3]. The European Centre for Disease Prevention and Control (ECDC) pointed out that the residents' age and frailty, heterogeneity of staff qualification, lack of personal protection equipment, and SARS-CoV-2-testing possibilities contributed to the high burden of care homes during COVID-19 [4]. Additionally, care homes with many residents, especially those with dementia, may perceive a higher burden due to the pandemic [5, 6]. Problems with implementing hygiene measures, the isolation of residents, and the fear of infection and its possible consequences caused a tense work environment [7, 8]. Therefore, a high psychosocial burden in care home staff was inevitable [3, 6, 9–11]. A systematic review and meta-analysis on the global psychosocial burden of health care professionals during COVID-19 showed 37%, 41%, and 45% prevalence rates for depression, anxiety, and stress [12]. In Germany, the most extensive study on psychosocial burden and working conditions of health care workers mainly in hospitals found a prevalence of 19% for depression and anxiety [13]. Though evidence for the psychosocial burden among health care workers during COVID-19 is vast, research focusing on care home workers is scarce. To our knowledge, there are three studies reporting prevalence rates of different psychopathological symptoms among care home workers; conducted in Italy, Spain, and the United States [14–16]. Riello and colleagues found prevalence rates of 22% and 39% for anxiety and Posttraumatic Stress Disorder (PTSD) among 1071 care home workers in Italy [14]. In Spanish care home workers, a prevalence of 49%, 59%, and 71% was found for depression, anxiety, and PTSD, respectively [15]. Further, 35.4% of care home workers in Michigan (United States) reported elevated rates of either PTSD, depression, anxiety, and/or stress [16]. Factors associated with increased psychosocial burden were female gender, knowledge about how to do the job, being a nurse, and contact to staff and/or residents with COVID-19. Moreover, support at work was found to be a protective factor. There are currently no studies evaluating the psychosocial burden of nurses in care homes during the first wave of the pandemic in Germany. Moreover, there is a lack of evidence on what structural factors of care homes and personal factors of nurses are associated with the COVID-19-related burden at work, stress, anxiety, and depression.

Our study aimed to investigate and determine associated factors of psychosocial burden among nurses working at care homes during Germany's first wave of the COVID-19 pandemic.

## Methods

### Study population

A retrospective online survey among nurses working in care homes was conducted between the 15^th^ of November 2020 and the 28^th^ of February 2021. The targeted time frame of the survey was between the 1^st^ of March 2020 and the 30^th^ of June 2020 in accordance with the first wave of the pandemic and the following ‘Lockdown’ in Germany. The survey is part of the cooperation project *COVID-Heim*, which aims to draw lessons from the pandemic for structural developments in the care home setting by combining various data sources in Germany. The sample consisting of 811 nurses was recruited online via social media. We invited all nurses working in care homes in Germany to participate in our online survey in relevant German facebook groups. The survey was provided by a secure web application for building and managing online surveys and databases – Research Electronic Data Capture System (REDCap). The questionnaire was opened 1,884 times and fully completed by 811 nurses. For the anonymous survey, we followed all relevant guidelines and regulations. The online survey was approved by the ethics committee of the Faculty of Medicine of the Charité – Universitäsmedizin Berlin (EA1/254/20).

### Measures

#### Characteristics of participants and affiliated care homes

The questionnaire included the following sociodemographic characteristics of the participants: age, gender, qualification (certified nurse, geriatric nurse, nurse in training, health care assistant, other), employment status (permanent, temporary), and risk factors for COVID-19 infections (e.g., >60 years, cardiovascular disease, diabetes mellitus, immunodeficiency, chronic obstructive pulmonary disease) which was taken from the COVID-19 Pandemic Mental Health Questionnaire (CoPaQ) [17]. The characteristics of the affiliated care homes included questions regarding size of care home (small [1-50 residents], medium [51-100 residents], large [>100 residents]), focus of care (e.g. dementia, mental illness, palliative care) and COVID-19 cases residents/staff (no COVID-19 cases, 1-20 COVID-19 cases, 11-20 COVID-19 cases, >20 COVID-19 cases).

#### COVID-19-related measures

##### Working demands since the pandemic

To evaluate the change in working needs since the coronavirus pandemic, we asked nurses how working demands changed during the COVID-19 pandemic since 1^st^ of March 2020, with three possible answer categories: 1) strongly/rather increased 2) did not change 3) rather/strongly decreased.

##### Satisfaction with COVID-19-related management of care home manager

We asked nurses whether they were satisfied with the COVID-19-related management of their care home manager between the time of 1^st^ of March until 30^th^ of June 2020 (e.g., implementation of protection and hygiene measures against the COVID-19, communication with staff); two answers were possible: 1) very/rather satisfied, 2) very/rather unsatisfied.

##### COVID-19-related burden at work

Nurses were asked to state to what extent the following possible COVID-19-related responsibilities at work were perceived as burden between 1^st^ of March until 30^th^ of June 2020 on a 4-point Likert scale ranging from 0) no burden to 3) extremely severe burden. Items were 1) The concern about the wellbeing of the residents; 2) Purchase and consumption of personal protection equipment; 3) Compliance with hygiene guideline of Robert Koch-Institute; 4) Concerns about COVID-19-infections among staff; 5) Concerns about COVID-19-infections among residents; 6) Implementation of protection measures (e.g., isolation of COVID-19-infected residents, contact precautions for relatives); 7) Small number of COVID-19-tests for residents; 8) Small number of COVID-19-tests for staff; 9) High expectations of relatives. The nine single items were adapted from Hower, Pfaff, and Pförtner [9]. We generated an index – ranging from 0 to 27 - by summing up all items answered. Higher results indicate more burden due to COVID-19 at work. The scale obtained a Cronbach's alpha score of 0.82 in our sample, which can be considered as ‘good’ [18].

##### COVID-19-related anxiety

Nurses were asked to state to what extent the following statements applied to them in the time from 1^st^ of March until 30^th^ of June 2020 on a 7-point Likert scale ranging from 0) does not apply at all to 7) fully applies. Items were 1) I was afraid to get infected with Corona; 2) I was afraid of the consequences of the Corona Pandemic on my life; 3) I was afraid of the consequences for my health if I got infected; 4) I was afraid of the consequences for the health of my relatives if I got infected; 5) I was afraid of the social consequences of Corona and 6) I was afraid of the economic consequences of Corona on my life. The six single items were adapted from Petzold and colleagues [19]. We generated an index - ranging from 0 to 36 - by summing up all item answers. Higher results indicate more COVID-19-related anxiety. The scale obtained a Cronbach's alpha score of 0.80 in the present sample, considered ‘good’ [18].

#### Stress, anxiety, and depression

##### Stress Scale (SDASS-21)

Stress symptoms were measured with the Stress Scale of the Depression Anxiety Stress Scale (DASS-21). The subscale consists of 7 items and asks for stress criteria on a 4-point Likert scale ranging from 0) did not apply to me at all to 3) applied to me very much/most of the time. The final scores - ranging from 0 to 21 - were multiplied by two [20, 21]. A score of 14 or less is considered ‘normal,’ 15-18 ‘mild,’ 19-25 ‘moderate,’ 26-33 ‘severe,’ and 34 or greater is considered ‘extremely severe’ [22]. The DASS-21 is widely used to study depression, anxiety, and stress in the general population and health care workers [12, 23]. In the present sample, the validated German version of the stress scale of the DASS-21 obtained a Cronbach’s Alpha of 0.88.

##### Generalized Anxiety Disorder-2 (GAD-2)

General anxiety symptoms were measured with the ultra-short 2-item version of the 7-item scale GAD-7. It incorporates the first two questions of the GAD-7, which are critical components of every anxiety disorder [24]. The score ranges from 0 to 6. A cut-off value of ≥ 3 was suggested to detect possible clinically relevant levels of anxiety symptoms. The GAD-2 is widely used to screen for general signs of anxiety, and its psychometric properties are well documented [25]. In the present sample, the Cronbach’s alpha of the GAD-2 was 0.80.

##### Patients-Health-Questionnaire-2 (PHQ-2)

Depressive symptoms were measured with the ultra-short 2-item version of the 9-item scale PHQ-9 [26]. It incorporates the first two questions of the PHQ-9. The score ranges from 0 to 6. A cut-off value of ≥3 was suggested to detect possible clinically relevant levels of depressive symptoms. The PHQ-2 has been widely used during the COVID-19 pandemic [12, 13]. In the present sample, the Cronbach’s alpha of the PHQ-2 was 0.82.

#### Social Relations at Work

Social Relations at work were measured by 3 subscales of the validated Copenhagen Psychosocial Questionnaire (COPSOQ) [27]. We used ‘support at work (4-item scale)’, ‘feedback (2-item scale)’ and ‘sense of community (2-item scale)’ All items were scored on a 5-point Likert scale ranging from 0) never/hardly ever to 100) always. In our sample, the 3 subscales obtained Cronbach’s alpha scores of 0.83, 0.69, and 0.88, respectively.

### Statistical analysis

Frequencies, percentages, means, and standard deviations for characteristics of participants and affiliated care homes were generated. Further, normal distribution was tested using the Shapiro-Wilk-Test. The Shapiro-Wilk-Test revealed that the data was not normally distributed. Therefore, relationships between Covid-19-related burden at work, stress, anxiety, depression, and categorical characteristics of participants and affiliated care homes were tested using the Mann-Whitney-U-Test or the Kruskal-Wallis-Test, respectively. We used Spearman correlation tests to test relationships between COVID-19-related burden at work, stress, anxiety, depression, and continuous variables. Finally, we used multiple regression analyses to determine factors associated with COVID-19-related burden at work, stress, anxiety, and depression. Analyses were performed using IBM SPSS Statistics for Windows, version 25.0 (IBM Corp., Armonk, NY). P values <.05 were considered to indicate statistical significance.

## Results

### Characteristics of nurses and affiliated care homes

In total, *N* = 811 nurses participated in the survey with a mean (±SD) age of 39.5 years (±10.7), the proportion of female nurses was 90.6% (*n* = 735), and 55.1% of the nurses (*n = 447)* stated to have at least one risk factor for a severe course of a COVID-19 infection. The majority of participants were geriatric nurses (72.1%, *n* = 585), 96.1% (*n* = 771) of the nurses had a permanent position in the care home they are working at, 77.6% of the nurses worked at medium or large care homes (*n* = 616) and 30.5% of the nurses confirmed dementia as a focus of care at their care home (*n* = 247). Additionally, most of the nurses reported COVID-19 cases among the residents and the staff (63.8%, *n* = 504; 72.4%. *n* = 567), respectively, at their care homes. Furthermore, 94.2% of the nurses confirmed working demands since the pandemic rather or strongly increased (*n* = 763), and 42.3% stated that they are rather or very unsatisfied with the COVID-19 management of their care home manager (*n* = 343). The mean (±SD) for COVID-19-related burden and COVID-19-related anxiety were 17.8 (±5.5) and 22.2 (±8.5), respectively. Table 1 shows the characteristics of nurses and affiliated care homes in detail.

**Table 1:** Characteristics of participants and affiliated care homes (*N* = 811)

	n	Valid %
***Characteristics of participants***		
*Gender*		
Female	735	90.6
Male	74	9.1
Divers	2	0.2
*Age (years)*		
17-39	439	54.1
40-67	372	45.9
Mean (S*D)*	39.5 (10.7)	
*Qualification*		
Certified Nurse	116	14.3
Geriatric Nurse	585	72.1
Nurse in Training	13	1.6
Health Care Assistant	50	6.2
Other	47	5.8
*Permanent position (yes)*	771	96.1
≥ *1 risk factor for a severe course of COVID-19*	447	55.1
**COVID-19-related measures**		
*Working demands since the pandemic*		
Strongly/rather increased	763	94.2
Did not change	31	3.8
Rather/strongly decreased	11	1.4
*Satisfaction with COVID-19 management of care home manager*		
Very/rather satisfied	467	57.7
Very/rather unsatisfied	343	42.3
*COVID-19-related burden at work (Mean, SD)*	17.8 (5.5)	
*COVID-19-related anxiety (Mean, SD)*	22.2 (8.5)	
**Stress (SDASS-21), Anxiety (GAD-2) & Depression (PHQ-2)**		
*Stress*		
Mean (SD)	16.7 (10.2)	
Score ≥ 19 (N, %)	308 (39.2)	
*Anxiety*		
Mean (SD)	2.2 (1.8)	
Score ≥ 3 (N, %)	294 (36.5)	
*Depression*		
Mean (SD)	2.5 (1.8)	
Score ≥ 3 (N, %)	332 (41.4)	
*Stress, Anxiety and/or Depression (N, %)*	466 (59.1)	
**Social Relations at work (COPSOQ)**		
*Support at work (Mean, SD)*	60.8 (27.4)	
*Feedback (Mean, SD)*	46.3 (28.6)	
*Sense of community (Mean, SD)*	72.0 (23.0)	
***Characteristics of affiliated care homes***		
*Size of care home*		
Small (1-50 residents)	177	22.3
Medium (51-100 residents)	350	44.1
Large (> 101 residents)	266	33.5
*Dementia as focus of care (yes)*	247	30.5
*COVID-19 cases residents (yes)*	504	63.8
*COVID-19 cases staff (yes)*	567	72.4.

### Prevalence of stress, anxiety, and depression

In the present sample, 39.2% of the nurses (*n* = 308) showed moderate to extremely severe stress symptoms according to the stress scale (SDASS-21); 36.5% (*n* = 294) and 41.4% (*n* = 332) of the nurses showed clinically relevant symptoms of Anxiety (GAD-2) and Depression (PHQ-2). Altogether, 59.1% (*n* = 466) of the nurses showed values above the cutoff on at least one of the three screening instruments used (SDASS-21, GAD-2, PHQ-2; see Table 1).

### Characteristics of nurses in relation with psychosocial burden

We further investigated the relationship between COVID-19-related burden at work, stress, anxiety, depression, and characteristics of participants and affiliated care homes (see Table 2). At first, nurses rather or very unsatisfied with COVID-19 management of their care home manager showed significantly higher scores on all 4 scales than those rather or very satisfied (p < .001). Moreover, we found that older nurses perceived a significantly higher COVID-19-related burden at work than younger nurses (p < .01). Also, nurses working in care homes with dementia as a focus of care perceived a significantly higher COVID-19-related burden at work than those who do not (p < .01). In addition to that, female nurses had significantly higher scores on the stress scale of the DASS-21 compared to their male counterparts (p < .05). Also, nurses with at least 1 risk factor for a severe course of COVID-19 showed significantly higher anxiety scores than those with no risk factors (p < .05). Finally, younger nurses (17-39 years) and nurses with at least 1 risk factor for a severe course of COVID-19 showed significantly higher scores of Depression compared to their counterparts (p < .01, p < .05). Correlations between scales can be found in the Additional file 1.

**Table 2:** Relationship between psychosocial burden and characteristics of participants and affiliated care homes

	COVID-19-related burden at work		Stress		Anxiety		Depression	
	Mdn (IQR)	***p***-value	Mdn (IQR)	***p***-value	Mdn (IQR)	***p***-value	Mdn (IQR)	***p***-value
**Characteristics of participants**								
*Gender*								
Female	18 (7.0)	.09	**16 (14.0)**	**<.05**	2 (2.0)	.06	2 (3.0)	.54
Male	17 (6.5)		**12 (18.5)**		1 (3.0)		2 (3.0)	
*Age (years)*								
17-39	**18 (7.0)**	**<.01**	16 (18.0)	.29	2 (3.0)	.48	**2 (3.0)**	**<.01**
40-67	**19 (8.0)**		16 (14.0)		2 (2.0)		**2 (2.0)**	
*Qualification*								
Certified Nurse	19 (9.0)		16 (16.0)		2 (2.0)		2 (2.0)	
Geriatric Nurse	18 (7.0)	.13	16 (15.0)	.95	2 (2.0)	.66	2 (3.0)	.49
Nurse in Training	20 (6.0)		18 (14.0)		3 (3.0)		3 (2.0)	
Health Care Assistant	17 (7.0)		16 (19.0)		2 (2.8)		2 (3.0)	
Other	21 (7.0)		17 (16.5)		2 (2.3)		2 (3.0)	
≥ *1 risk factor for a severe course of COVID-19*								
Yes	18 (7.0)	.58	18 (14.0)	.20	**2 (3.0)**	**< .05**	**2 (3.0)**	**<.05**
No	18 (8.0)		16 (16.0)		**2 (2.0)**		**2 (2.0)**	
*Satisfaction with COVID-19 management of care home manager*								
Very/rather satisfied	**18 (7.0)**	**<.001**	**14 (14.0)**	**<.001**	**2 (2.8)**	**<.001**	**2 (2.0)**	**<.001**
Very/rather unsatisfied	**19.5 (7.0)**		**18 (14.0)**		**2 (3.0)**		**3 (2.0)**	
**Characteristics of affiliated care homes**								
*Size of care home*								
Small (1-50 residents)	18 (7.0)	.19	18 (16.0)	.53	2 (2.8)	.67	2 (2.0)	.53
Medium (51-100 residents)	18 (7.0)		16 (16.0)		2 (2.0)		2 (3.0)	
Large (> 101 residents)	18 (8.0)		16 (16.0)		2 (3.0)		2 (3.0)	
Dementia as focus of care								
Yes	**19 (8.0)**	**<.01**	16 (16.0)	.61	2 (2.5)	.88	2 (3.0)	.65
No	**18 (8.0)**		16 (14.0)		2 (2.0)		2 (3.0)	
*COVID-19 cases residents*								
Yes	18 (7.0)	.29	16 (14.0)	.24	2 (3.0)	.06	2 (3.0)	.25
No	18 (8.0)		16 (16.0)		2 (3.0)		2 (3.0)	
*COVID-19 cases staff*								
Yes	18 (7.0)	.20	16 (16.0)	.80	2 (2.0)	.47	2 (3.0)	.91
No	18 (8.0)		16 (15.0)		2 (2.3)		2 (3.0)	

*Note.* Mdn = Median. IQR = Interquartile range. Significant values are shown in bold type.

### Multivariate associations between characteristics of nurses and psychosocial burden

Multivariate results indicate that COVID-19-related burden at work was significantly associated with qualification: health care assistants perceived significantly less COVID-19-related burden at work compared to certified nurses (β = -.132, 95% CI: -.21, -.04; see Table 3). Furthermore, COVID-19-related burden at work was significantly associated to the satisfaction with the COVID-19 management of the care home manager with those being rather or very unsatisfied perceived significantly higher burden (β = .10, 95% CI: .02, .17).

**Table 3:** Multiple Regression Analysis on Covid-19-related burden at work, Stress (SDASS-21), Anxiety (GAD-2) and Depression (PHQ-2)

	Covid-19-related burden at work		Stress		Anxiety		Depression	
	β (95% CI)	***p***-value	β (95% CI)	***p***-value	β (95% CI)	***p***-value	β (95% CI)	***p***-value
**Characteristics of participants**								
*Gender (Male)*	-.06 (-.12; .01)	﻿.12	-.04 (-.11; .03)	.29	-.06 (-.14; .01)	.10	-.00 (-.07; .07)	.98
*Age (years)*	.05 (-.02; .12)	.14	**-.08 (-.16; -.01)**	**<.05**	-.04 (-.12; .04)	.29	-.07 (-.14; .00)	.06
*Qualification*								
Nurse *[Reference]*								
Geriatric Nurse	-.05 (-.14; .04)	.31	-.00 (-.10; .09)	.97	.08 (-.02; .18)	.12	**.11 (.02; .21)**	**<.05**
Nurse in Training	.01 (-.07; .08)	.85	-.02 ( -.09; .06)	.63	.04 (-.04; .12)	.31	.03 (-.04; .11)	.39
Health Care Assistant	**-.13 (-.21; -.04)**	**<.01**	.06 (-.03; .14)	.17	.07 (-.02; .15)	.14	.08 (-.01; .16)	.07
Other	.04 (-.04; .12)	.31	-.00 (-.08; .08)	.98	.00 (-.08; .09)	.94	.02 (-.06; .10)	.63
≥ *1 risk factor for severe course of COVID-19 (yes)*	.03 (-.04; .10)	.38	.01 (-.06; .08)	.84	.06 (-.02; .13)	.12	.03 (-.05; .09)	.51
**COVID-19-related measures**								
*Satisfaction with COVID-19 management of care home manager (very/rather unsatisfied)*	**.10 (.02; .17)**	**<.05**	.01 (-.07; .10)	.73	.00 (-.08; .08)	.99	.03 (-.05; .11)	.49
*COVID-19-related burden at work*	**-**		**.19 (.11; .28)**	**<.001**	**.16 (.08; .25)**	**<.001**	**.15 (.06; .23)**	**﻿<.001**
*COVID-19-related anxiety*	**.45 (.38; .52)**	**<.001**	**.26 (.18; .34)**	**<.001**	**.18 (.10; .26)**	**<.001**	**.15 (.07; .23)**	**<.001**
**Social Relations at work (COPSOQ)**								
*Support at work*	-.08 (-.18; .02)	.13	**-.18 (-.29; -.07)**	**<.001**	**-.16 (-.27; -.05)**	**<.01**	**-.25 (-.35; -.14)**	**<.001**
*Feedback*	.00 (-.09; .09)	.98	.02 (-.08; .11)	.71	.05 (-.05; .15)	.32	.03 (-.07; .12)	.59
*Sense of community*	.01 (-.07; .10)	.76	**-.09 (-.18; -.01)**	**<.05**	**-.11 (-.20; -.03)**	**<.05**	**-.11 (-.20; -.02)**	**<.05**
**Characteristics of affiliated care homes**								
*Size of Care Home*								
Small (1-50 residents*) [Reference]*								
Medium (51-100 residents)	-.01 (-.10; .08)	.82	-.08 (-.18; .01)	.08	-.07 (-.16; .03)	.17	-.03 (-.12; .06)	.48
Large (> 100 residents)	-.00 ( -.09; .09)	.96	**-.10 (-.19; -.01)**	**<.05**	-.04 (-.14; .05)	.39	-.03 (-.12; .07)	.61
*Dementia as focus of care (yes)*	**.07 (.00; .14)**	**<.05**	-.07 (-.14; .01)	.07	-.02 (-.09; .05)	.57	-.04 (-.12; .03)	.25
*COVID-19 cases residents (yes)*	-.01 (-.10; .08)	.85	**.10 (.00; .19)**	**<.05**	.09 (-.01; .18)	.08	.06 (-.04; .15)	.23
*COVID-19 cases staff (yes)*	.05 (-.04; .14)	.29	-.08 (-.17; .02)	.10	-.04 (-.14; .05)	.39	-.04 (-.13; .06)	.46
***R***^***2***^ *(adjusted R*^*2*^*)*	**.27** (.25)		**.25** (.23)		**.18** (.15)		**.21** (.18)	

*Note.* β = standardized coefficient. CI = confidence interval. Significant values are shown in bold type.

COVID-19-related burden at work was also significantly associated to COVID-19-related anxiety (β = .45, 95% CI: .38, .52) and working in a care home with dementia as focus of care (β = .07, 95% CI: .00, .14). There were significantly negative associations between stress and the age of the nurses (β = -.08, 95% CI: -.16, -.01), support at work (β = -.18, 95% CI: -.29, -.07), sense of community (β = -.09, 95% CI: -.18, -.01) and size of the care home. Nurses working in large care homes were significantly less stressed compared to nurses working in small care homes (β = -.10, 95% CI: -.19, -.01). In contrast, there was a significantly positive association between stress and COVID-19-related burden at work (β = .19, 95% CI: .11, .28), COVID-19-related anxiety (β = .26, 95% CI: .18, .34) and COVID-19 cases among residents (β = .10, 95% CI: .00, .19). Anxiety was significantly positively associated with COVID-19-related burden at work (β = .16, 95% CI: .08, .25) and COVID-19-related anxiety (β = .18, 95% CI: .10, .26), while it was significantly negative associated with support at work (β = -.16, 95% CI: -.27, -.05), and sense of community (β = -.11, 95% CI: -.20, -.03). Depression was significantly associated with qualification, with geriatric nurses showing more depressive symptoms compared to certified nurses (β = .11, 95% CI: .02, .21), with COVID-19-related burden at work (β = .15, 95% CI: .06, .23), and COVID-19-related anxiety (β = .15, 95% CI: .07, .23). A significantly negative association with depression was shown with support at work (β = -.25, 95% CI: -.35, -.14), and a sense of community (β = -.11, 95% CI: -.20, -.02; see Table 3). There were no significant associations between the presence of at least 1 risk factor for a severe course of COVID-19, feedback and COVID-19 cases among staff with one of the four scales.

## Discussion

The present study aimed to investigate stress, anxiety, depression, and COVID-19-related burden at work for nurses in care homes during the first wave of COVID-19 in Germany. Further, we wanted to determine factors associated with the psychosocial burden of nurses in care homes. We have found that COVID-19-related burden was lower in health care assistants, was associated with higher COVID-19-related anxiety, and with dementia as a focus of care. Stress was significantly higher in younger healthcare workers, was associated with higher COVID-19-related anxiety, with increased COVID-19-related work burden and lack of support and sense of community at work. Stress was higher where more COVID-19 cases were diagnosed, but was less in larger care homes. Anxiety and depression were associated with higher COVID-19-related anxiety, with increased COVID-19 related work burden, lack of support, and sense of community at work.

In Comparison to evidence from the Organisation of Economic Co-operation and Development (OECD), providing data on the long-term care workforce of 38 countries worldwide, the present study sample was slightly younger, higher qualified, and more often in a permanent working status [28]. Moreover, 55% of the nurses reported at least one risk factor for a severe course of COVID-19, which is in line with a previous study by Greene and Gibson, which found a similar percentage (50%) of COVID-19 risk factors in the long term care workforce of the United States [29].

Our study supports evidence from other countries that the COVID-19 pandemic had a high impact on working demands and workload of care home staff [7, 8, 11, 30]. In the presen sample, the vast majority of nurses reported an increased working demand since the pandemic, with 42% stating that they were unsatisfied with the COVID-19 management of their care home manager. Prior research showed that care home staff often did not feel involved in decision-making processes and did not feel heard by the management of their care homes. A lack of communication between management and staff caused uncertainty and distress about handling the pandemic and implementing measures to prevent infections [31–33].

The present study showed increased prevalence rates of stress, anxiety, and depression (59.1%). About 39% of the nurses showed moderate to extremely severe stress symptoms, 36.5% showed clinically significant anxiety, and 41.4% showed clinically significant depression. The prevalence rates found are different from those in Italy, Spain, and Michigan (USA) [14–16]. In Northern Italy, a prevalence rate of 22% for anxiey was found; 59% for anxiety and 49% for depression were found in Spain. Our findings may be due to the different impacts of the pandemic on countries and structural differences in care homes. Additionally, prior studies included all staff working in care homes, not exclusively nursing staff. Nevertheless, the prevalence rates found in the present study are similar to the meta-analysis on the global psychosocial burden of health care workers during COVID-19 [12]. After analyzing 83 studies, a pooled prevalence of 37% for depression, 41% for anxiety, and 45% for stress among health care workers worldwide were found. Compared to prevalence rates for depression and anxiety found in German healthcare workers in general (19%) and the general population (25%), prevalence rates for depression and anxiety were higher in the present sample [13, 19].

Besides, our findings showed that COVID-19-related burden at work (e.g., lack of personal protection equipment, a small number of COVID-19-Tests for residents and staff) was positively associated with the dissatisfaction with COVID-19 management of care home management, COVID-19- related anxiety, and dementia as a focus of care. This is in line with previous research showing that there was uncertainty about implemented hygiene measures due to a lack of communication and information and not being included in decision-making processes by care home management during the pandemic [8, 31–33]. A higher burden for care home staff working with residents with dementia, in general, was a concern even before the pandemic [34]. Compared to certified nurses, health care assistants were significantly less burdened due to COVID-19 at work. Prior research showed these ambiguous findings. Some results also imply that health care assistants are less prone to burnout and stress in general and due to COVID-19 than certified nurses [35–38]. Other studies did not show any differences or even the opposite [39, 40].

Stress, anxiety, and depression were associated with COVID-19-related burden at work, COVID-19-related anxiety, less support at work, and less sense of community. Additionally, stress was negatively associated with age and working in a large care home and positively associated with working in a care home with COVID-19 cases among residents. Though we hypothesized that working in a large care home may be associated with increased psychosocial burden, our results showed the opposite regarding stress symptoms. A possible explanation could be that larger care homes had more possibilities to implement specific hygiene measures, i.e., isolate infected residents, divide areas, and build different `care groups` where only certain nurses would regularly take care of the same residents.

Our study has strengths and limitations. Strengths include a large sample size and detailed, well-validated, and widely used assessments and questionnaires to generate prevalence rates of stress, anxiety, and depression for german nursing staff in care homes. Certain limitations need to be considered. At first, the study sample was recruited online via social media only. Thus, nursing staff with limited access to the internet or web-enabled devices might not have participated in this survey. Further, our study sample was slightly younger with higher qualifications than Germany's usual long-term care workforce [41], raising the issue of selection bias. Both problems may limit generalizability to the entire workforce of nursing staff in german care homes. Moreover, although we took the number of COVID-19 cases into account, our data does not differentiate for regions or COVID-19 hotspots, though there was variation in how much certain areas of Germany were affected by the pandemic. Finally, the retrospective nature of our survey may have biased answers as subsequent events may have influenced the answering of nursing staff.

## Conclusion

In summary, high psychosocial burden among nursing staff in care homes in Germany has been shown. These results add new information into the understudied area of care home staff burden and the COVID-19 pandemic. The results could yield strategies that needed to be addressed to help and support nursing staff during a crisis and beyond. Regular psychosocial counseling and programs for the prevention of mental illnesses could be a strategy to buffer the high demands of the nursing job and to prevent the worsening of already existing distress symptoms. Furthermore, social support and a sense of community are essential to cope with an extraordinary circumstance like the pandemic. Regular team meetings, counseling with the care home management, and involving staff in decision-making processes could help strengthen the bond between staff and management and buffer future crises.

## Supplementary Information

**Additional file 1:** Relationship between Psychosocial burden and Social Relations at work (COPSOQ).

## Data Availability

The dataset used for the current retrospective survey is available from the corresponding author on reasonable request.
