# Supplementary material for: Psychosocial burden and associated factors among nurses in care homes during the COVID-19 pandemic: findings from a retrospective survey in Germany
Source: BMC Nurs. 2022 Feb 10;21:41. doi: 10.1186/s12912-022-00807-3 (PMC8830035; doi:10.1186/s12912-022-00807-3)
Supplement: Supplementary file 1 — Additional file 1: Relationship between Psychosocial burden and Social Relations at work (COPSOQ). [file 12912_2022_807_MOESM1_ESM.docx]

| **Relationship between Psychosocial burden and Social Relations at work (COPSOQ)** | | | | | | | | | | |
| --- | --- | --- | --- | --- | --- | --- | --- | --- | --- | --- |
|  |  |  | 1 | 2 | 3 | 4 | 5 | 6 | 7 | 8 |
| **COVID-19-related measures** | |  |  |  |  |  |  |  |  |  |
| 1 | COVID-19-related burden at work |  | 1 |  |  |  |  |  |  |  |
| 2 | COVID-19-related anxiety |  | **.43**** | 1 |  |  |  |  |  |  |
| **Stress (SDASS-21), Anxiety (GAD-2) & Depression (PHQ-2)** | | | | |  |  |  |  |  |  |
| 3 | Stress |  | **.32**** | **.36**** | 1 |  |  |  |  |  |
| 4 | Anxiety |  | **.26**** | .**29**** | **.68**** | 1 |  |  |  |  |
| 5 | Depression |  | .**24**** | **.26**** | .**60**** | **.71**** | 1 |  |  |  |
| **Social Relations at work (COPSOQ)** | | | |  |  |  |  |  |  |  |
| 6 | Support at work |  | **-.17**** | **-.13**** | **-.28**** | **-.26**** | **-.35**** | 1 |  |  |
| 7 | Feedback |  | **-.10**** | -.04 | **-.17**** | **-.13**** | **-.24**** | .**65**** | 1 |  |
| 8 | Sense of Community |  | -.07 | **-.08*** | **-.22**** | **-.24**** | **-.29**** | **.53**** | **.40**** | 1 |

*Note.* significant values are shown in bold type. *p < .05, ** p < .01 and *** p < .001
